# Precise Identification of Prostate Cancer from DWI Using Transfer Learning

**DOI:** 10.3390/s21113664

**Published:** 2021-05-25

**Authors:** Islam R. Abdelmaksoud, Ahmed Shalaby, Ali Mahmoud, Mohammed Elmogy, Ahmed Aboelfetouh, Mohamed Abou El-Ghar, Moumen El-Melegy, Norah Saleh Alghamdi, Ayman El-Baz

**Affiliations:** 1 Bioengineering Department, University of Louisville, Louisville, KY 40292, USA; islam-cis@mans.edu.eg (I.R.A.); ahmed.shalaby@louisville.edu (A.S.); ahmahm01@louisville.edu (A.M.); aselba01@louisville.edu (A.E.-B.); 2 Faculty of Computers and Information, Mansoura University, Dakahlia 35516, Egypt; melmogy@mans.edu.eg (M.E.); elfetouh@mans.edu.eg (A.A.); 3 Radiology Department, Urology and Nephrology Center, University of Mansoura, Dakahlia 35516, Egypt; maboelghar@mans.edu.eg; 4 Electrical Engineering Department, Assiut University, Assiut 71515, Egypt; moumen@aun.edu.eg; 5 College of Computer and Information Science, Princess Nourah Bint Abdulrahman University, Riyadh 11564, Saudi Arabia

**Keywords:** prostate cancer, transfer learning, ALexNet, VGGNet, ADC maps

## Abstract

**Background and Objective:** The use of computer-aided detection (CAD) systems can help radiologists make objective decisions and reduce the dependence on invasive techniques. In this study, a CAD system that detects and identifies prostate cancer from diffusion-weighted imaging (DWI) is developed. **Methods:** The proposed system first uses non-negative matrix factorization (NMF) to integrate three different types of features for the accurate segmentation of prostate regions. Then, discriminatory features in the form of apparent diffusion coefficient (ADC) volumes are estimated from the segmented regions. The ADC maps that constitute these volumes are labeled by a radiologist to identify the ADC maps with malignant or benign tumors. Finally, transfer learning is used to fine-tune two different previously-trained convolutional neural network (CNN) models (AlexNet and VGGNet) for detecting and identifying prostate cancer. **Results:** Multiple experiments were conducted to evaluate the accuracy of different CNN models using DWI datasets acquired at nine distinct b-values that included both high and low b-values. The average accuracy of AlexNet at the nine b-values was 89.2±1.5% with average sensitivity and specificity of 87.5±2.3% and 90.9±1.9%. These results improved with the use of the deeper CNN model (VGGNet). The average accuracy of VGGNet was 91.2±1.3% with sensitivity and specificity of 91.7±1.7% and 90.1±2.8%. **Conclusions:** The results of the conducted experiments emphasize the feasibility and accuracy of the developed system and the improvement of this accuracy using the deeper CNN.

## 1. Introduction

Prostate cancer is a major health problem, especially in western countries. For example, this disease is the second leading cause of mortality among males in the United States [1]. In 2020, the number of new prostate cancer cases and the number of deaths caused by prostate cancer among Americans are expected to be 191,930 and 33,330, respectively [1]. Currently, the definitive technique of diagnosing prostate cancer is transrectal ultrasound (TRUS)-guided biopsy. However, biopsy is an invasive procedure that can miss up to 30% of cancers [2]. In order to minimize the errors of detecting prostate cancer by TRUS-guided biopsy, many alternatives, such as magnetic resonance imaging (MRI)-guided biopsy, have been investigated [3].

Recently, MRI has evolved in its capabilities of detecting prostate cancer in addition to its use in guiding biopsies for better accuracy. Multiple MRI sequences, which include T2-weighted MRI, diffusion-weighted imaging (DWI), and dynamic contrast-enhanced MRI (DCE-MRI), are used in various clinical tasks, such as active surveillance, localization, and determining the stage of prostate cancer [4,5]. However, detecting and localizing prostate cancer from MRI data is a challenging task. As large volumes of MRI data, from different protocols and sometimes from different scanners, have to be analyzed, these variations can lead to inter-observer differences. Computer-aided detection (CAD) systems can help physicians make objective and fast decisions. These systems can also enhance the quantitative evaluation of prostate cancer.

Developing MRI-based CAD systems for identifying prostate cancer has become a subject of active research [6]. For instance, Viswanath et al. [7] examined the performance of multiple classical classifiers and their bagging and boosting ensembles using a multi-institutional T2-weighed MRI dataset of 85 subjects. These classifiers were fed with radiomic features and their performance was evaluated using the receiver operating characteristic (ROC) curve. The highest average area under the curve (AUC) was obtained by the boosted quadratic discriminant analysis. Riccardo et al. [8] found that the accuracy of targeted biopsy improved by 13.2% in case of combining physician analysis of multiparametric MRI with CAD results. Rampun et al. [9] compared the accuracy of eleven classifiers using a T2-weighed MRI dataset of 45 subjects. Their system employed feature selection on a feature space of size 215 to select the best discriminating features. The highest resulting accuracy was 85.5%. More detailed and profound literature review can be found in the recent survey by Gao et al. [10].

Although existing systems have achieved satisfactory results, these systems base their diagnosis on handcrafted features that are validated on small datasets. The good empirical design of these handcrafted features determines their accuracy. An alternative approach for handcrafted features is to learn the discriminating features automatically.

Deep learning structures, especially convolutional neural networks (CNN), are able to automatically learn multiple levels of features from data in a hierarchical manner [11]. These structures have achieved accurate results in multiple computer vision tasks [12,13,14,15,16] as well as lesions detection tasks [17]. Ishioka et al. [18], developed a prostate cancer CAD algorithm that aimed to reduce the variation in the interpretation of T2-weighted MRI. They used histogram smoothing to convert the 12-bit intensity data from the original T2-weighted MR images into 8-bit images. These images were normalized and subjected to data augmentation to increase the employed training data. The detection of prostate cancer was obtained using a CNN architecture that combined both U-net and ResNet50. Their algorithm resulted in an AUC of 0.65. Mehrtash et al. [19] evaluated the performance of CNN with different MRI modalities. The highest performance achieved by their system in terms of AUC was 0.8. This performance was achieved by including zonal information of tumors with DWI and DCE-MRI. Yang et al. [20] proposed a system for detecting and localizing the presence of prostate cancer from T2-weighted MRI and apparent diffusion coefficient (ADC) images. Their system used an individual CNN for each of the used two modalities to produce a response map indicating the malignancy likelihood of each pixel. An average pooling is performed before the last convolutional layer of each CNN to obtain a feature vector. The feature vectors from each modality are concatenated and used as input into a support vector machine (SVM). A sensitivity of 46% and 97% was achieved at 0.1 and 10 false positives per normal case. Le et al. [21] integrated a similarity cost function with the CNN cost function to better fuse ADCs with T2-weighted MRI. The authors investigated multiple data augmentation techniques, multiple CNN architectures, and multiple fusion schemes to find out the combination that can lead to the best accuracy. The final differentiation between malignant and benign tissues was based on the integration between the results of CNN and the results of handcrafted features using an SVM classifier. An accuracy of 91.5% was achieved. Wang et al. [22] found that optimizing the steps of prostate segmentation, multi-modal registration and cancer localization in a joint manner can reduce the computation burdens of optimizing each step individually. Moreover, this joint optimization improves the accuracy by reducing the accumulation of errors over the different steps. Song et al. [23] developed a patch-based CNN system for differentiating between malignant and benign prostate lesions from multiparametric MRI. Their proposed CNN model consisted of three blocks of layers before the final fully-connected (FC) layers. Each block consisted of three convolutional layers followed by a dropout layer and a max-pooling layer. Their model resulted in sensitivity and specificity of 87% and 90.6%, respectively. Hosseinzadeh et al. [24] developed 2D-UNet model to produce maps of prostate zones. In their system, the inclusion of zonal information improved the average sensitivity at three different false positives by 5%.

Schelb et al. [25] used a dataset of two modalities (T2-weighted MRI and DWI) to train a U-Net classifier. Their system showed that the performance of a U-Net classifier is similar to the clinical assessment. Xu et al. [26] developed a system to detect prostate lesions using a residual network. Their system resulted in an accuracy of 93%. Yuan et al. [27] developed a system for classifying prostate cancer from three MRI modalities. Their systems employed three CNNs. Each CNN was fed with a different modality to learn discriminative features. Their system resulted in an accuracy of 86.9%. Chen et al. [28] tuned two pre-trained CNNs, namely, InceptionV3 and VGG-16, using PROSTATEx challenge dataset. An AUC of 0.83 was obtained using VGG-16. Abbasi et al. [29] tuned a pre-trained CNN model(GoogLeNet) using a prostate cancer MRI dataset. They compared GoogLeNet with other classifiers, such as, decision tree and SVM. They found that GoogLeNet outperformed these conventional classifiers. A recent survey by Wildeboer et al. [30] listed more than 80 CAD systems for diagnosing prostate cancer. In this survey, the different CAD systems were categorized according to the employed imaging modalities and the employed classifiers.

Training deep architectures with huge numbers of parameters from scratch requires large amounts of data. Typically, the amount of data in the medical domain is small when compared to that of natural images used in conventional computer vision applications. Training deep architectures with small amounts of data can lead to overfitting, which means the network can correctly classify the data used for training but is not able to correctly classify new data (i.e., the network does not generalize well). Moreover, if the amount of medical data is sufficient, annotating this data by multiple experts to prepare it for training can be an impeding factor. In order to overcome these limitations, this work modifies previously-trained CNNs and fine-tunes them for detecting and identifying prostate cancer. The process of fine-tuning such deep networks can be done with small datasets.

The main contribution of this work does not depend on which deep learning network is used; the main contribution is representing the input data in a different form (ADC maps) to be more separable to achieve acceptable accuracy, no matter which deep learning network is used. To prove this point, AlexNet, which is a deep learning network that has only five convolutional layers, is used in the beginning. AlexNet achieved an average accuracy of 89.2±1.5% at the nine b-values. For further validation, VGGNet, which has more layers that increase its learning ability, is then used. VGGNet achieved an average accuracy of 91.2±1.3% at the nine b-values. The ADC maps used in the proposed system are calculated at both low and high b-values. This enables the proposed system to capture both blood perfusion and water diffusion for an accurate diagnosis of prostate cancer. The b-value is a measure of the degree of diffusion weighting employed to generate diffusion-weighted images. It is a parameter that relies on the timing and strength of the used gradient pulses. The details of this process are discussed in the following sections.

## 2. Methods and Materials

The DWI datasets used in this work were collected from 37 subjects (16 benign and 21 malignant) using two distinct scanners (1.5 Tesla (T) scanner and 3T scanner) at nine distinct b-values (100, 200, …, 800, 1000 s/mm^2^). The acquisition parameters of the 1.5 T scanner in axial plane were: TE = 84.6 ms, TR = 8000 ms, Bandwidth = 142.86 kHz, FOV = 34 cm, slice thickness = 3 mm, inter-slice gap = 0 mm, voxel size = 1.25×1.25×3.00 mm^3^. The 3T DW images were acquired in the transverse plane. The acquisition parameters of the second 3T scanner were: TR = 4300–4800 ms, TE = 75–88 ms, acquisition matrix = 128×126, reconstruction matrix = 256×256, FOV = 20 cm. The scanning was done using full-echo and full-k-space. However, a SENSE acceleration factor of 2 was used, thus skipping over every-other line in k-space. Excitation and read-out were spectral fat-suppressed, standard 2D multi-slice 90–180 spin-echo Stejskal-Tanner DWI, with single-shot echo-planar-imaging read-out. A single diffusion encoding direction of [1,1,1] was used (i.e., X,Y,Z gradient channels were on simultaneously) to obtain minimal TE at the maximum b-value. Each b-value was averaged 7 times. Big delta = 47 ms and little delta = 15 ms. All the cases involved in this study performed MRI when there was a clinical suspicion of malignancy. The final diagnosis of the cases was established by biopsy that was carried out after MRI. Therefore, the ground truth of diagnosis of all subjects was based on the results of the biopsy. From the malignant cases, 234 slices were labelled manually by a radiologist as having malignant tumors. The analysis and labeling of all the cases were performed in a slice-wise manner. A similar number, 236, of DW slices from benign cases were chosen to create a balanced dataset of malignant and benign slices. These slices represent all the DW slices of 13 benign cases in addition to 5 slices from the remaining 3 benign cases. The ADC maps of the DWI datasets were calculated, as will be explained in the following subsection. The corresponding 470 ADC maps of the labelled DW slices were used to train and test the performance of the developed model, as explained in the following sections.

**Figure 1:**
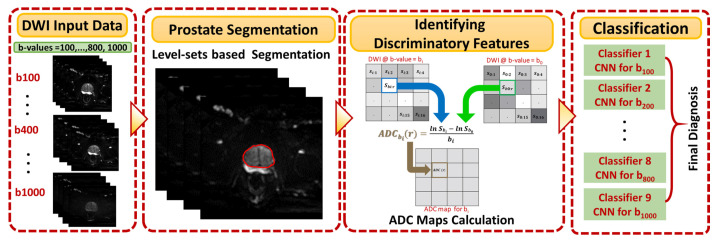
Overall workflow of the proposed model showing the DWI input data at nine b-values and its three basic steps, which are prostate segmentation, calculation of ADC maps as discriminating features, and the identification of slices with tumor using previously-trained CNN models.

**Figure 2:**
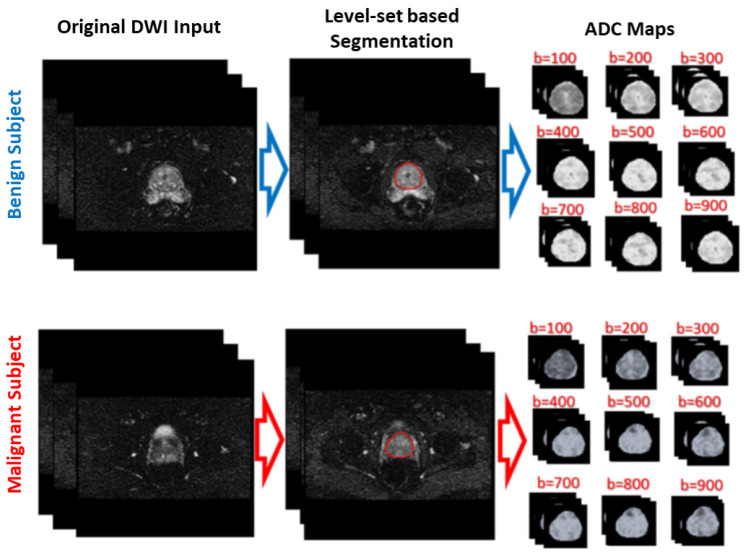
Illustration of the first two processing steps of the proposed framework on two different cases (one benign and one malignant).

Figure 1 shows the general framework of the developed CAD system for detecting and identifying prostate cancer, which incorporates three main steps: prostate segmentation, identifying discriminatory features, and identifying slices with prostate cancer. Prostate segmentation integrates three types of features using non-negative matrix factorization (NMF) to guide the evolution of a level set model. These features are shape-priors of prostates, intensities of voxels and spatial features of neighboring voxels. The effect of incorporating each of these features into the accuracy of the used segmentation approach is explained in detail in [31]. Appearance, shape and spatial information were extracted for each voxel. NMF was used to reduce and make the combined features more separable. Curvature and Euclidean distances between the reduced features (test subject) to the centroids of NMF classes (calculated from training subjects) were used to estimate the voxel-wise guiding force of the level set. If the voxel belongs to the prostate, the level set grows. Otherwise, the level set shrinks. More details about the used segmentation approach can be found in [31]. The second step was identifying discriminatory features, which can discriminate malignant from benign cases. In this study, ADC volumes of the segmented prostates were estimated and used for this purpose. By nature, the prostate is a small organ compared to other organs. To be sure that the proposed model would capture the features that discriminate between malignant tumors and benign tumors, the ADC features were calculated only from the prostate region. This ensured that the system learned the features related only to prostate cancer. The first two processing steps of the proposed framework are illustrated for two different cases (one benign and one malignant) in Figure 2.

**Figure 3:**
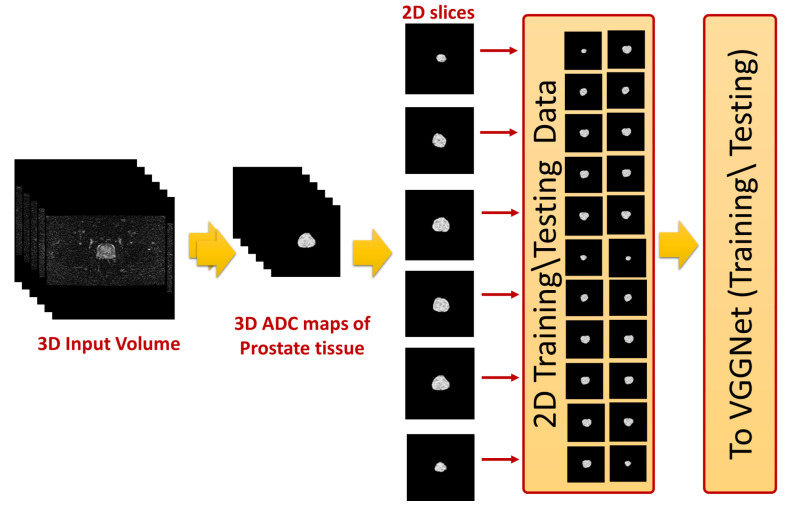
Illustration of slice-wise analysis of ADC volumes.

The 2D cross sections that constitute these ADC volumes were extracted and used as input to the employed CNN-based model. This process is shown in Figure 3. The final step was identifying slices with tumors using a previously-trained CNN model. In the following subsections, the details of estimating the ADC maps and the use of these maps to fine-tune CNN models for identifying prostate cancer are presented.

### 2.1. Identifying Discriminatory Features

Currently, DWI is one of the promising modalities utilized for the identification of prostate cancer. DWI is a functional MRI modality similar to DCE-MRI. However, what distinguishes DWI is its fast acquisition time, as there are no contrast agents used. The problem with contrast agents is the potential harm they cause to patients who have kidney disorders. DWI depends on the differences in the motion of water molecules inside the body to create images. DW images visualize and quantify this microscopic motion [32]. The molecules’ motion is random, and there is a positive correlation between the level of randomness and the loss in the signal, which is given by [33]:

(1) $$S_{d}∼e^{-b\times ADC},$$

where *b* is a parameter that relies on the timing and strength of the used gradient pulses, and ADC is a measure of the magnitude of water molecules’ diffusion within the tissues. The utilization of gradient pulses gives rise to enhanced diffusion sensitivity compared to the steady state gradients [34].

The intensities of pixels in a slice acquired at a specific b-value ($S_{b}$) are equal to the intensities of the congruent pixels of the baseline slice (*b* = 0 s/mm^2^) lowered by the signal loss defined in Equation (1). These intensities are given by:

(2) $$S_{b}=S_{0}\times e^{-b\times ADC}.$$

There is a negative relationship between the cellular density of a tissue region and its ADC values, as regions with dense cells restrict the mobility of water molecules. Since the quality of DWI is low, a large number of researchers choose to utilize the quantitative ADC maps computed from DWI to identify prostate cancer. The reason for the discriminating capabilities of ADCs is that malignant prostate tissues have smaller ADC values than healthy or benign tissues. The following equation shows that two DW images are required to calculate an ADC map:

(3) $$ADC=-\frac{lnS_{b_{1}}-lnS_{0}}{b_{1}}.$$

The first image is collected at a specific b-value ($b_{1}$ > 0 s/mm^2^) while the second is collected at $b_{0}$ (*b* = 0 s/mm^2^). Another justification for employing ADC maps in this study is that the calculation of ADC maps is not sensitive to the used magnetic field strength [35]. This is suitable for the DWI datasets used in this study, as they were collected by two scanners that have different magnetic field strengths. Moreover, integrating handcrafted features with the automatically-learned features by CNNs can improve accuracy [36]. Since each ADC map represents the difference between two DW images, these maps are themselves images. Therefore, they can be used as input to the CNN model instead of the DW images. As these maps have better discriminating capabilities, their use improves the accuracy of the system.

### 2.2. Identification of Prostate Cancer

In this study, two different CNN models were used for prostate cancer identification. There are multiple advantages of using CNN over traditional neural networks. First, CNNs typically contain a larger number of layers than traditional neural networks. Augmenting the number of layers allows CNN to learn high levels of abstraction as the first layers learn primitive components while end layers use these learned primitive features to form the high-level features. The process of learning the features is done automatically by CNNs. Second, CNN takes both 2D images and 3D volumes directly as inputs without the need to convert these inputs into vectors, as in the case of neural networks. This preserves the inputs’ spatial information. Third, the network connections and hence the network parameters of CNNs are fewer than the network connections in similar traditional neural networks. This reduction simplifies and expedites the training process of CNNs [37,38].

In this work, DW slices that contained tumors were labeled by a radiologist. The ADC maps that correspond to these labeled DW slices were divided into two groups. The first group contained ADC maps with malignant tumors, and the second group contained ADC maps from benign subjects. The number of ADC maps in the benign group was 236, and the number of ADC maps in the malignant group was 234. These ADC maps were used to train and evaluate two different previously-trained CNN models, which are AlexNet [39] and VGGNet [40].

ALexNet expects an input image of size 227×227×3, whereas the size of an input image for VGGNet was 224×224×3. The sizes of the calculated ADC maps were the same as the sizes of the corresponding DW images, which were 256×256 for the 1.5T images and 144×144 for the 3T images. To make these ADC maps suitable as inputs for the employed CNN models, the ADC maps of the larger sizes were center cropped, while the ADC maps of smaller sizes were zero padded. Then, each of these ADC maps was concatenated along the depth dimension to generate a three-channel image, which was the expected input to each of the employed CNN models.

**Figure 4:**
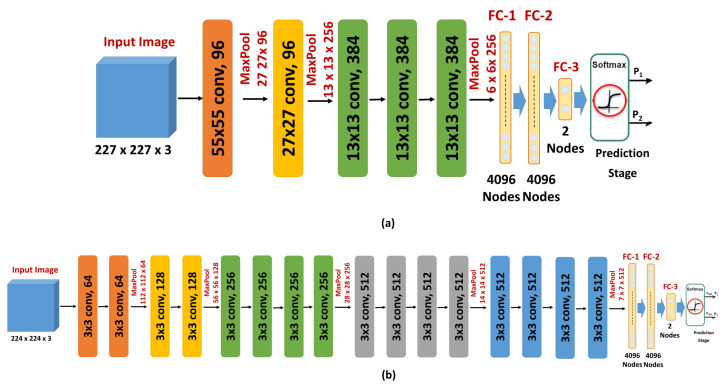
Illustration of the two different CNNs used in this study, (**a**) ALexNet, and (**b**) VGGNet.

Since the number of ADC maps is considered small for training and evaluating a CNN model from scratch, as training such deep structures from scratch with a small dataset leads to overfitting, a transfer learning model was adopted in this study. The idea of transfer learning is to modify a network that is trained to solve a certain problem and use it to solve a new problem in a different domain. The training of the original network is typically done with millions of images from the original domain. The advantage of transfer learning is that the adoption of this previously-trained network to solve a new problem requires far fewer images from the new domain. This is done by replacing the last few layers, including the output layer of the original network, with new layers appropriate to the new problem. In this work, the original output layer of either AlexNet or VGGNet, which assigns its input image to one of 1000 categories, was replaced with an output layer that classified its input ADC maps into either benign or malignant (Figure 4).

The introduction and success of AlexNet have revolutionized the use of CNNs for multiple classification tasks. AlexNet is a CNN that contains five convolutional layers and three FC layers (Figure 4a). The network depth has a remarkable influence on its accuracy as the accuracy of AlexNet drops by 2% in the case of removing any of the five convolution layers. Rectified linear units (ReLUs) [41] are employed as activation functions by AlexNet. There are two main advantages of using ReLUs. First, ReLUs are saturation-free even in case of unnormalized inputs. Second, the training time of CNNs with ReLUs is shorter than the training time of CNNs with saturating activations (e.g., sigmoid). AlexNet is trained with a large dataset of natural images (ImageNet). This dataset contains more than one million images that belong to one thousand categories. To reduce the classification error, AlexNet employs overlapping pooling and response normalization [39]. AlexNet employs dropout [42] and data augmentation to overcome the overfitting issue. Two different forms of data augmentation were used: intensity alteration and transformations (translations and horizontal reflections) [39].

VGGNet is another deep CNN that is trained using ImageNet dataset. The main goal of developing VGGNet is to evaluate the effect of the network depth on the accuracies of CNNs. To achieve this goal, the developers examined five different network architectures with different depths, while the other parameters are fixed for a fair comparison. The input image is processed by a sequence of convolution layers, max-pooling layers [43], FC layers, and a softmax layer. VGGNet uses ReLU non-linearity activation. The number of convolutional layers of the different architectures ranges from 8 to 16 layers. The numbers of pooling layers and FC layers are 5 and 3, respectively. These numbers do not differ across the different architectures. The pooling layers follow some, but not all, of the convolutional layers. The convolution layers use kernels of small fixed receptive fields of 3×3 to lower the number of parameters. The pooling layers use fixed windows of 2×2 and a stride of 2. The number of filters of the first convolutional layer is 64. When a pooling layer is used, the number of filters of the following convolutional layer is doubled until the number of kernels reaches 512 [40].

In this work, the deepest architecture was used. This architecture has 19 layers with weights (16 convolutional layers and 3 FC layers) (Figure 4b). This architecture has 144 million parameters. This large number of parameters increases the training time of VGGNet, especially when compared with other CNNs with smaller numbers of parameters, such as AlexNet (60 million parameters) and GoogLeNet (4 million parameters). However, the performance of VGGNet in many transfer learning tasks is better than GoogLeNet [44]. Both AlexNet and VGGNet were optimized using stochastic gradient descent with momentum and the loss function was cross entropy. The other training parameters were the following: number of epochs = 50, learning rate = 0.0001, momentum = 0.9, mini-batch-size = 10, L_2_ regularization = 0.0001. The basic architecture and configuration parameters of both the original and the fine-tuned AlexNet and VGGNet are summarized in Table 1.

## 3. Results

Multiple experiments were conducted to test the performance of the developed system and to compare its performance with other modern machine-learning classifiers. In the first experiment, 70% of the ADC maps of both the malignant and benign cases at each b-value were used for fine-tuning an AlexNet-based model. The other 30%, which represent 71 ADC maps from benign cases and 70 ADC maps with malignant tumors, were employed to evaluate the accuracy of the tuned model. The ADC slices used for training were from different patients to the ADC slices used for testing to avoid any correlation that could exist between ADC slices of the same patient. The results of this experiment at each b-value are shown in Table 2.

In a similar experiment, 80% of the ADC maps were used for tuning an AlexNet-based model. The remaining 20% or 94 ADC maps were used to evaluate the accuracy of the tuned model. The reason behind this 80:20 division of the dataset was to satisfy the Pareto principle. The results of this experiment are reported in Table 3.

Similarly, in another experiment, 70% of the ADC maps of both the benign and malignant cases at each b-value were randomly chosen for tuning an AlexNet-based model. The other 30% of the ADC maps were used to evaluate the accuracy of the tuned model. Since the ADC maps used for evaluating the accuracy of the system at each b-value were chosen randomly, this experiment was repeated 10 times at each b-value. To ensure the stability of the reported results, the mean accuracy of the 10 experiments conducted at each b-value, in addition to the mean sensitivity and the mean specificity, are listed in Table 4.

In another experiment, 10-fold cross validation was applied using the 470 ADC maps. Each fold contained 47 ADC maps. Nine folds were used for training whereas the remaining fold was used for testing the system. This operation was repeated 10 times with the change of the testing fold each time. The performance of the 10-fold cross validation using AlexNet at the different b-values is reported in Table 5.

In order to evaluate the effect of the depth of the used CNNs on the resulting accuracy, an experiment was conducted. In this experiment, 70% of the ADC maps were used for tuning a deeper CNN (VGGNet), while the remaining 30% were used to evaluate the trained model. VGGNet has 16 convolutional layers in comparison to five convolutional layers in AlexNet. The performance using VGGNet is listed in Table 6. As can be noticed, the use of the deeper network improves the accuracy at all b-values except for b-value = 500 s/mm^2^. The highest improvement in the accuracy is at b-value = 600 s/mm^2^. The use of VGGNet leads to an average improvement of 1.97% in the accuracy.

The time required for training VGGNet model is much longer than the time required for training the AlexNet model. For example, the time required for fine-tuning AlexNet in the first experiment was 5 min and 25 s, whereas the time required for fine-tuning the deeper CNN (VGGNet) in the similar experiment was about 97 min. The proposed models were developed using MATLAB 2017b. The training of the proposed model was performed using a workstation with a graphics processing unit (GPU) of type NVIDIA Quadro K1200.

In a similar experiment, 80% of the ADC maps were used for tuning a VGGNet-based model. The remaining 20% or 94 ADC maps were used to evaluate the accuracy of the tuned model. The results of this experiment are reported in Table 7.

In another experiment, 10-fold cross validation is applied using VGGNet at the different b-values. The results of this experiment are reported in Table 8.

In order to show the merits of CNNs over classical machine-learning classifiers, the performance of CNN is compared to the performance of SVM with both linear and quadratic kernels. The models of SVMs were trained using 70% of the ADC maps and evaluated using the remaining 30% of the ADC maps. The inputs to the SVMs were the same as the inputs to the CNNs. The inputs to the SVMs were the ADC maps (raw data). In order to be used as input to the SVMs, each ADC map has to be transformed into a vector. This vector represents a row in the data matrix used to train the SVM. The results of these SVMs are reported in Table 9. The high performance of the CNN models highlights the importance of the inputs’ spatial information that is preserved in the case of CNNs. However, the inputs’ spatial information is lost in the case of conventional models such as SVMs.

**Figure 5:**
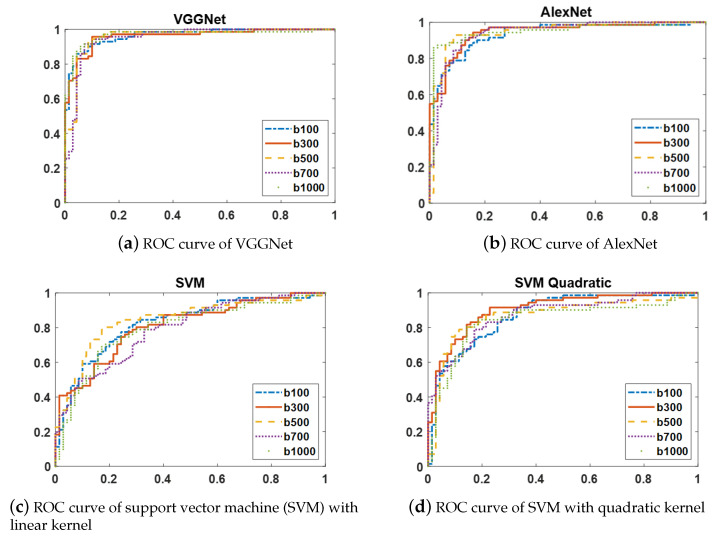
ROC curves at five b-values(100, 300, 500, 700, and 1000 s/mm^2^) of four different classifiers: (**a**) VGGNet, (**b**) AlexNet, (**c**) SVM with linear kernel, and (**d**) SVM with quadratic kernel. As these figures show, CNN-based models (VGGNet and AlexNet) result in higher AUCs than the two variants of SVMs with linear and quadratic kernels at the distinct b-values.

The ROC curves of two CNN models (AlexNet and VGGNet) and two variants of SVMs with linear and quadratic kernels are shown in Figure 5. Since the ROC curves of each of these classifiers at the distinct b-values have similar shapes, the ROC curves at only five b-values are displayed to simplify the figures.

An experiment was conducted to compare the performance of the proposed approach to one of the state-of-the-art CNNs, which is GoogLeNet [45]. This CNN was the winner of the ImageNet challenge in 2014. This deep network consists of 22 layers. However, the number of its parameters is reduced dramatically due to the use of the Inception module and the removal of the FC layers. The resulting performance of GoogLeNet is listed in Table 10. The performance results of GoogLeNet are close to the results of both AlexNet and VGGNet. These results boosts the feasibility of the transfer learning in diagnosing prostate cancer.

## 4. Discussion

In this study, a transfer learning model is adopted to detect and identify prostate cancer. When the employed CNN models were originally trained using natural images, they used conventional techniques such as, data augmentation and dropout in order to reduce the effect of overfitting. The combination of both conventional overfitting handling techniques and transfer learning can minimize the effect of overfitting.

The proposed system starts with segmentation to limit the region-of-interest (ROI) to the prostate region only. In this system, prostate segmentation is performed using level set due to its capability to provide continuous segmented object. However, any segmentation approach can be integrated with the proposed system, as long as, it provides a continuous segmented object. For example, Comelli et al. [46], presented a fast deep learning network, namely efficient neural network (ENet), for prostate segmentation from T2-weighted MRI. ENet is initially used for image segmentation tasks in self-driving cars where hardware availability is limited and the accuracy is critical for user safety. In this study [46], ENet is trained using a dataset of 85 subjects and results in a dice similarity coefficient of 90.89%.

Several studies suggested that the use of DWI acquired at higher b-values are preferable for accurate detection and diagnosis of prostate cancer [47,48,49,50,51]. This study shows that the use of ADC maps calculated at lower b-values results in an accuracy close to the accuracy of the ADC maps calculated at higher b-values. There is a slight accuracy increase for the ADC maps calculated at higher b-values. This accuracy increase is more obvious in the case of using a less-deeper CNN (AlexNet). One of the advantages of using ADC maps is that they are insensitive to the magnetic field strengths of the used scanners (1.5T or 3T) [35]. The ADC maps used in this study were calculated from DWI acquired with 1.5T and 3T scanners at nine b-values. The results show that the dependence on ADC maps can also mitigate the differences in the accuracy between higher and lower b-values, especially in the case of using deeper CNN models.

The developed approach performs slice-wise analysis. However, the proposed framework is generic and can perform both slice-wise analysis and prostate-zonal analysis based on how the model is trained. Since the system shows good performance in slice-wise analysis, the authors did not investigate it on zonal analysis. Investigating the performance of the system in prostate-zonal analysis is a good potential for future work.

According to the literature, a sensitivity and a positive predictive value of 80% and 87%, have been reported for men with high prostate-specific antigen (PSA) values using positron emission tomography/computed tomography (PET/CT) [52,53].

According to Sun et al. [54], the performance of deep learning networks increases logarithmically based on the size of the training data. One way to improve the performance in the case of limited data is to conduct multiple experiments and choose the best results. In this work, to obtain the best performance, 10-fold cross validation was used to obtain almost the same performance, no matter which fold was used for training and which fold was used for testing.

The use of two different CNN models in this work shows that the depth of the CNN model positively affects the performance of the system. However, much longer processing times are required to train the deeper architectures. The results of this study show that the use of a deeper CNN (VGGNet) increases the accuracy of prostate cancer detection more than the less-deep CNN (AlexNet). However, this accuracy is still far from perfect. Examining the effect of using much deeper CNN models, such as ResNet [55], can be a potential future work. Moreover, in this system, prostate cancer identification from DWI acquired at nine b-values was investigated. This investigation can be extended by performing a statistical analysis of the used b-values to select the best minimal combination of b-values that lead to the best accuracy. Choosing a minimal combination of b-values will reduce both the acquisition time of DWI and the computational complexities. Another area of potential future work could be the use of artificial intelligence optimization techniques on a combination of imaging markers and clinical markers (such as PSA) to optimize prostate cancer management.

## 5. Conclusions

In conclusion, this paper presents a CAD system for prostate cancer detection and identification from DWI. The identification of prostate cancer is achieved using two previously-trained CNN models (AlexNet and VGGNet) that are fed with the estimated ADC maps of the segmented prostate regions. The conducted experiments show that the use of previously-trained CNN models for detecting prostate cancer is feasible. These previously-trained CNN models learn the discriminatory features automatically. The results section shows that CNN models outperform conventional models. The accuracy of conventional models depends on the good design of the used handcrafted features.

## Figures and Tables

**Table 1:** Basic architecture and configuration parameters of both AlexNet and VGGNet, where Conv. means convolutional, FC means fully-connected, SGDM means stochastic gradient descent with momentum, and cross entr. means cross entropy.

	AlexNet		VGGNet	
	Original	Fine-Tuned	Original	Fine-Tuned
No. of training images	>1 million	329–423	>1 million	329–423
Size of input images	227×227	227×227	224×224	224×224
No. of output categories	1000	2	1000	2
No. of Conv. layers	5	5	16	16
FC layers	3	3	3	3
Optimizer	SGDM	SGDM	SGDM	SGDM
Loss function	cross entr.	cross entr.	cross entr.	cross entr.

**Table 2:** Performance of AlexNet at 9 b-values using 141 ADC maps.

b-Value	Accuracy%	Sensitivity (Recall)%	Specificity%	Precision%
100 s/mm^2^	86.52	84.29	88.73	88.06
200 s/mm^2^	90.07	85.71	94.37	93.75
300 s/mm^2^	88.65	85.71	91.55	90.91
400 s/mm^2^	88.65	88.57	88.73	91.18
500 s/mm^2^	91.49	91.43	91.55	91.43
600 s/mm^2^	89.36	88.57	90.14	89.86
700 s/mm^2^	87.94	85.71	90.14	89.55
800 s/mm^2^	90.07	90.00	90.14	90.00
1000 s/mm^2^	90.07	87.14	92.96	92.06

**Table 3:** Performance of AlexNet at 9 b-values using 94 randomly chosen ADC maps.

b-Value	Accuracy%	Sensitivity (Recall)%	Specificity%	Precision%
100 s/mm^2^	87.23	87.23	87.23	87.23
200 s/mm^2^	90.43	89.36	91.49	91.30
300 s/mm^2^	89.36	89.36	89.36	89.36
400 s/mm^2^	89.36	91.49	87.23	87.76
500 s/mm^2^	90.43	91.49	89.36	89.58
600 s/mm^2^	91.49	89.36	93.62	93.33
700 s/mm^2^	88.33	89.36	87.23	87.50
800 s/mm^2^	91.49	93.62	89.36	89.80
1000 s/mm^2^	89.36	93.62	85.11	86.27

**Table 4:** Average Performance of AlexNet at 9 b-values using 141 randomly chosen ADC maps and repeating the experiment 10 times.

b-Value	Accuracy%	Sensitivity%	Specificity%
100 s/mm^2^	87.52±0.01	89.14±0.04	85.92±0.04
200 s/mm^2^	89.29±0.01	88.71±0.04	89.86±0.03
300 s/mm^2^	89.57±0.02	89.00±0.03	90.14±0.03
400 s/mm^2^	89.57±0.02	89.71±0.03	89.44±0.04
500 s/mm^2^	89.22±0.03	88.29±0.04	90.14±0.04
600 s/mm^2^	88.01±0.03	88.00±0.07	88.03±0.05
700 s/mm^2^	89.72±0.02	89.29±0.04	90.14±0.03
800 s/mm^2^	89.63±0.03	88.57±0.05	90.70±0.03
1000 s/mm^2^	90.43±0.02	90.86±0.04	90.00±0.04

**Table 5:** 10-fold cross validation of AlexNet at 9 b-values.

b-Value	100	200	300	400	500	600	700	800	1000
1st fold	93.62	87.23	93.62	93.62	91.49	89.36	91.49	91.49	87.23
2nd fold	78.72	82.98	78.72	82.98	87.23	82.98	85.11	80.85	78.72
3rd fold	91.49	95.74	91.49	89.36	93.62	93.62	95.74	93.62	91.49
4th fold	95.74	97.87	97.87	93.62	93.62	95.74	97.87	97.87	91.49
5th fold	87.23	89.36	91.49	91.49	89.36	93.62	87.23	91.49	91.49
6th fold	85.11	80.85	82.98	82.98	87.23	80.85	85.11	87.23	89.36
7th fold	91.49	93.62	89.36	97.87	95.74	93.62	93.62	95.74	93.62
8th fold	89.36	87.23	87.23	89.36	89.36	89.36	87.23	89.36	89.36
9th fold	93.62	91.49	91.49	93.62	91.49	93.62	89.36	91.49	91.49
10th fold	82.98	85.11	85.11	87.23	87.23	85.11	87.23	87.23	85.11
Average%	88.94	89.15	88.94	90.21	90.64	89.79	90.00	90.64	88.94

**Table 6:** Performance of VGGNet at 9 b-values using 141 randomly chosen ADC maps.

b-Value	Accuracy%	Sensitivity (Recall)%	Specificity%	Precision%
100 s/mm^2^	90.07	88.57	91.55	91.18
200 s/mm^2^	92.20	92.86	91.55	91.55
300 s/mm^2^	90.07	90.00	90.14	90.00
400 s/mm^2^	90.07	90.00	90.14	90.00
500 s/mm^2^	90.78	91.43	90.14	90.14
600 s/mm^2^	93.62	92.86	94.37	94.20
700 s/mm^2^	90.07	92.86	87.32	97.84
800 s/mm^2^	92.20	92.86	91.55	91.55
1000 s/mm^2^	91.49	92.86	90.14	90.28

**Table 7:** Performance of VGGNet at 9 b-values using 94 randomly chosen ADC maps.

b-Value	Accuracy%	Sensitivity (Recall)%	Specificity%	Precision%
100 s/mm^2^	91.49	87.23	95.74	95.35
200 s/mm^2^	90.43	89.36	91.49	91.30
300 s/mm^2^	91.49	91.49	91.49	91.49
400 s/mm^2^	90.43	87.23	93.62	93.18
500 s/mm^2^	92.55	89.36	95.74	95.45
600 s/mm^2^	94.68	91.49	97.87	97.73
700 s/mm^2^	90.43	87.23	93.62	93.18
800 s/mm^2^	92.55	89.36	95.74	95.45
1000 s/mm^2^	92.55	93.62	91.49	91.67

**Table 8:** 10-fold cross validation of VGGNet at 9 b-values.

b-Value	100	200	300	400	500	600	700	800	1000
1st fold	85.11	91.49	89.36	91.49	95.74	95.74	95.74	95.74	95.74
2nd fold	82.98	89.36	89.36	87.23	89.36	89.36	89.36	91.49	87.23
3rd fold	89.36	91.49	85.11	93.62	91.49	91.49	95.74	97.87	91.49
4th fold	93.62	91.49	89.36	93.62	97.87	97.87	97.87	93.62	97.87
5th fold	89.36	93.62	93.62	89.36	89.36	91.49	89.36	91.49	87.23
6th fold	89.36	80.85	87.23	87.23	82.98	85.11	89.36	89.36	82.98
7th fold	80.85	91.49	91.49	95.74	97.87	97.87	93.62	95.74	95.74
8th fold	87.23	97.87	93.62	91.49	97.87	87.23	89.36	93.62	85.11
9th fold	93.62	95.74	97.87	97.87	97.87	95.74	93.62	95.74	93.62
10th fold	78.72	80.85	89.36	82.98	85.11	85.11	80.85	85.11	87.23
Average%	87.02	90.43	90.64	91.06	92.55	91.70	91.49	92.98	90.43

**Table 9:** Performance of SVMs with linear kernel (lin. ker.) and quadratic kernel (quad. ker.) at 9 b-values using 141 randomly chosen ADC maps, where Acc. means accuracy, Sen. means sensitivity, and Spec. means specificity.

	SVM with Lin. Ker.			SVM with Quad. Ker.		
**b-Value**	**Acc. %**	**Sen. %**	**Spec. %**	**Acc. %**	**Sen. %**	**Spec. %**
100 s/mm^2^	75.18	65.71	84.51	78.01	71.43	84.51
200 s/mm^2^	75.89	72.86	78.87	78.72	78.57	78.87
300 s/mm^2^	73.05	58.57	87.32	82.27	77.14	87.32
400 s/mm^2^	68.79	75.71	61.97	80.85	88.57	73.24
500 s/mm^2^	79.43	82.86	76.06	82.27	81.43	83.10
600 s/mm^2^	73.76	68.57	78.87	80.14	77.14	83.10
700 s/mm^2^	68.79	71.43	66.20	80.14	80.00	80.28
800 s/mm^2^	71.63	74.29	69.01	81.56	87.14	76.06
1000 s/mm^2^	73.76	70.00	77.46	81.56	82.86	80.28

**Table 10:** Performance of GoogLeNet at 9 b-values using 141 ADC maps.

b-Value	Accuracy%	Sensitivity%	Specificity%
100 s/mm^2^	85.82	85.71	85.92
200 s/mm^2^	90.78	85.71	95.77
300 s/mm^2^	87.23	85.71	88.73
400 s/mm^2^	87.23	82.86	91.55
500 s/mm^2^	87.94	90.00	85.92
600 s/mm^2^	88.65	88.57	88.73
700 s/mm^2^	90.07	91.43	88.73
800 s/mm^2^	88.65	85.71	91.55
1000 s/mm^2^	89.36	90.00	88.73

## Data Availability

The data that support the findings of this study are available from the corresponding author upon reasonable request.
